# Factors Associated with the Perception of Speed among Recreational Skiers

**DOI:** 10.1371/journal.pone.0132002

**Published:** 2015-06-29

**Authors:** Friedrich Brunner, Gerhard Ruedl, Martin Kopp, Martin Burtscher

**Affiliations:** Department of Sport Science, University of Innsbruck, Innsbruck, Austria; McMaster University, CANADA

## Abstract

**Background:**

Skiers have to differ between slow to moderate and fast skiing speed to determine their skiing style according to the ISO 11088 standard for setting binding release values. Despite existing evidence that males ski significantly faster than females, no sex-specific factor was inserted into the ISO 11088 standard.

**Objective:**

To evaluate factors potentially associated with the perception of individual skiing speed among recreational skiers.

**Methods:**

Skiing speeds of 416 adult skiers (62% males,) were measured with a radar speed gun. Skiers were interviewed about their age, sex, skill level, risk taking behaviour and helmet use. Finally, skiers had to rate their perceived speed on one out of three speed categories (fast, moderate, slow).

**Results:**

The measured mean speed of this cohort was 48.2±14.3 km/h (30.0±8.9 mph). A total of 32%, 52%, and 16% of skiers perceived their actual speed as fast, moderate and slow, respectively. Mean speed differed significantly between the 3 speed categories with a mean of about 53.5±13.7 km/h (33.2±8.5 mph) for fast, 47.6±14.0 km/h (29.6±8.7 mph) for moderate, and 39.4±12.2 km/h (24.5±7.6 mph) for slow skiing, respectively. Sex (η^2^ = .074), skill level (η^2^ = .035) and risk taking behavior (η^2^ = .033) showed significant differences of skiing speeds with regard to the 3 categories of speed perception (all p < .001) while age groups and ski helmet use did not. Males, more skilled skiers and risky skiers perceived their actual speed as fast, moderate and slow, when skiing up to 10 km/h (6 mph) faster compared to females, less skilled and cautious skiers.

**Conclusion:**

The perception of skiing speed as fast, moderate or slow depends on sex, skill level, and risk taking behaviour. These findings should be considered when discussing the introduction of a sex factor into the ISO 11088 standard for setting binding release values.

## Introduction

Recreational alpine skiing is one of the most popular winter sports enjoyed yearly by several hundred million people worldwide [1]. Although the injury risk of less than 2 injuries per 1000 ski days [2,3] seems low, the total number of injuries per year remains high because of the huge population at risk. In recreational alpine skiing, severe injuries are often related to excessive speed [4,5]. Studies measuring speeds on ski slopes with a radar speed gun reported a mean skiing speed of 45–48 km/h (28–30 mph) depending on several factors [6,7]. In the study by Ruedl et al. [7], younger age, male sex, higher skill level, and a riskier behavior were found to be independent factors for higher speeds on ski slopes. In addition, a recently published study demonstrated that skiers underestimated their measured speed by 8% on average and that skiing speed, age, sex, skill level, and risk taking behavior seem to influence the ability to estimate the actual speed accurately [8]. Also important, however, seems the perception of the individual speed as fast, moderate or slow skiing speed considering that according to the ISO 11088 standard [9] for setting of binding release values, skiers have to differ between slow to moderate and fast skiing speeds to determine their skiing style. Beside skiing style, also age, height, and weight and ski shoe sole length of the skier were considered for the binding setting values. A correct binding setting is important because a too high binding setting puts skiers at risk for not releasing a ski during an accident while a too low binding setting can result in a more aggressive skier losing a ski at high speed due to an inadvertent release, both resulting in severe injuries. Although skiing speed depends on sex [6,7] and the proportion of binding non-release among female skiers with knee injuries is clearly higher compared to males [10], no sex-specific factor was inserted into the ISO 11088 standard [9] for binding release values. Albeit important for binding setting values and injury prevention, knowledge of the perception of skiing speed in recreational skiing seems incomplete at the moment. Therefore, this study aimed at answering the questions, how fast skiers move when they perceive their skiing speed slow, moderate or fast, and how this depends on sex, age, and various other factors potentially influencing skiing speed of recreational skiers.

## Materials and Methods

The data set of this study was also used in previously published articles [7,8,11] and the retrospective data analysis was approved by the Institutional Review Board Sport Science Innsbruck. The sample and the methods were described in detail in a previous work [8]. Briefly, speed measurements of skiers were performed with a radar speed gun on four ski slopes of medium difficulty in the winter season 2008/2009 [8]. Measured subjects were stopped and invited to participate in this study. Inclusion criterion for this study was an age older than 17 years. More than 90% of these subjects agreed to participate and gave their informed consent for the interview. Age, sex, ski helmet use, self-reported skill level according to Sulheim et al. [12] and self-reported risk taking behavior (cautious vs. risk taking) according to Ruedl et al. [13] were recorded for this study. With regard to the self-reported skill level (expert, advanced, intermediate or beginner), skiers were divided into more skilled (advanced and experts) and into less skilled (beginners and intermediates) persons. Finally, skiers had to rate their perceived speed on a five point Likert scale (very fast, fast, moderate, slow, very slow).

Due to a low number of skiers who perceived their skiing speed as very slow (n = 5) and very fast (n = 16), we decided to use only 3 different speed perception categories (slow, moderate, fast). Age was classified into 4 age groups (≤30, 31–40, 41–50, >50 years). Frequencies were evaluated by calculating percentages and the proportions (odds) of male to female skiers, and the odds ratio (OR) of more skilled to less skilled skiers, and differences between frequencies were evaluated with χ^2^-methods.

Differences of mean skiing speed between and within the 3 speed categories according to sex, age group, skill level, risk taking behavior and ski helmet use were evaluated by using factorial ANOVA (GLM). For the interpretation of differences, 2-tailed p-values ≤0.05 were considered for statistical significance and η^2^ = .01, .10 and .25 for small, medium and large effect size, respectively.

## Results

In total, 416 adult skiers (62% males,) with a mean age of 41.8±13.3 years, mean height of 175.2±8.8 cm, and mean weight of 75.1±14.2 kg participated in this study. Regarding nationality, 38.9% were Austrians, 47.6% were Germans, and 13.5% were from other countries. Mean measured speed of all participants was 48.2±14.3 km/h (30.0±8.9 mph). A total of 32.2%, 52.2%, and 15.6% of skiers perceived their actual speed as fast, moderate and slow, respectively.

### Frequency distributions within the 'slow', 'moderate' and 'fast' perception groups depending on sex, age, self-assessment of skill level, risk taking behavior and helmet use

**Fig 1:**
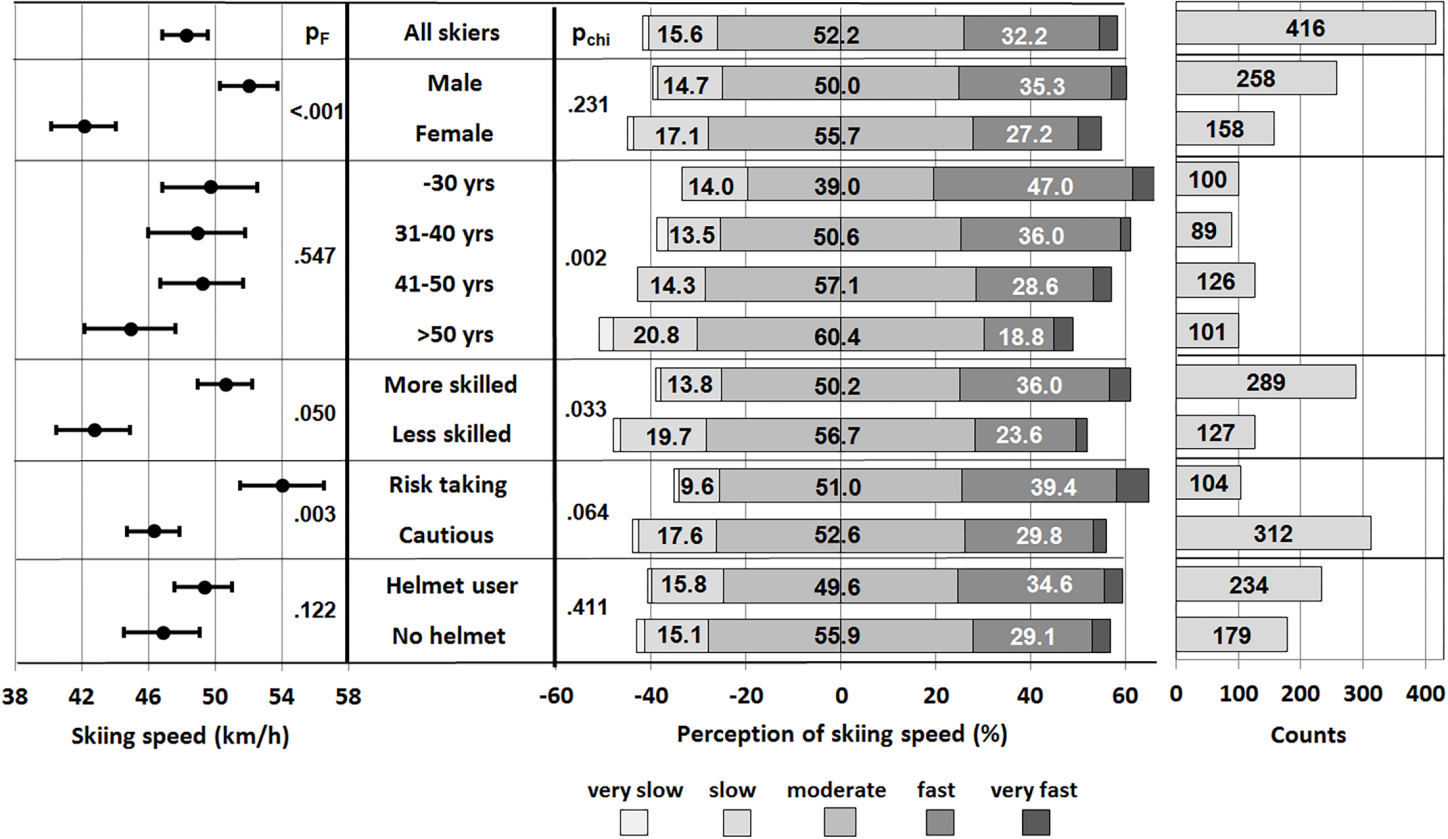
Error bars (mean ± 95% CI) of measured speed (total and according to factors sex, age, self-assessment of skiing skill level, risk taking behavior and helmet use) and diverging stacked bar charts showing the proportions (%) of speed perception groups ‘slow’, ‘moderate’ and ‘fast’. p_F_ the significance of F for the main effect of a factor on skiing speed. p_chi_ the significance of χ^2^ for contingency between factor and speed perception.

Frequency distributions of skiing speed perception depending on the factors analyzed are shown in the middle part of Fig 1. Chi square tests showed significant differences between age groups (p = .002) and between skill levels (p = .033). In addition a trend was found for risk taking behavior (p = .064). Differences of frequency distributions are mainly apparent in the 'fast' perception group, where younger (≤30 yrs: 47.0% vs. >50 yrs: 18.8%), more skilled (36.0% vs. less skilled: 23.6%) and risk taking skiers (39.4% vs. cautious: 29.8%) showed higher frequencies.

### Proportions of male to female skiers for less and more skilled skiers considering the factor perception of skiing speed

**Fig 2:**
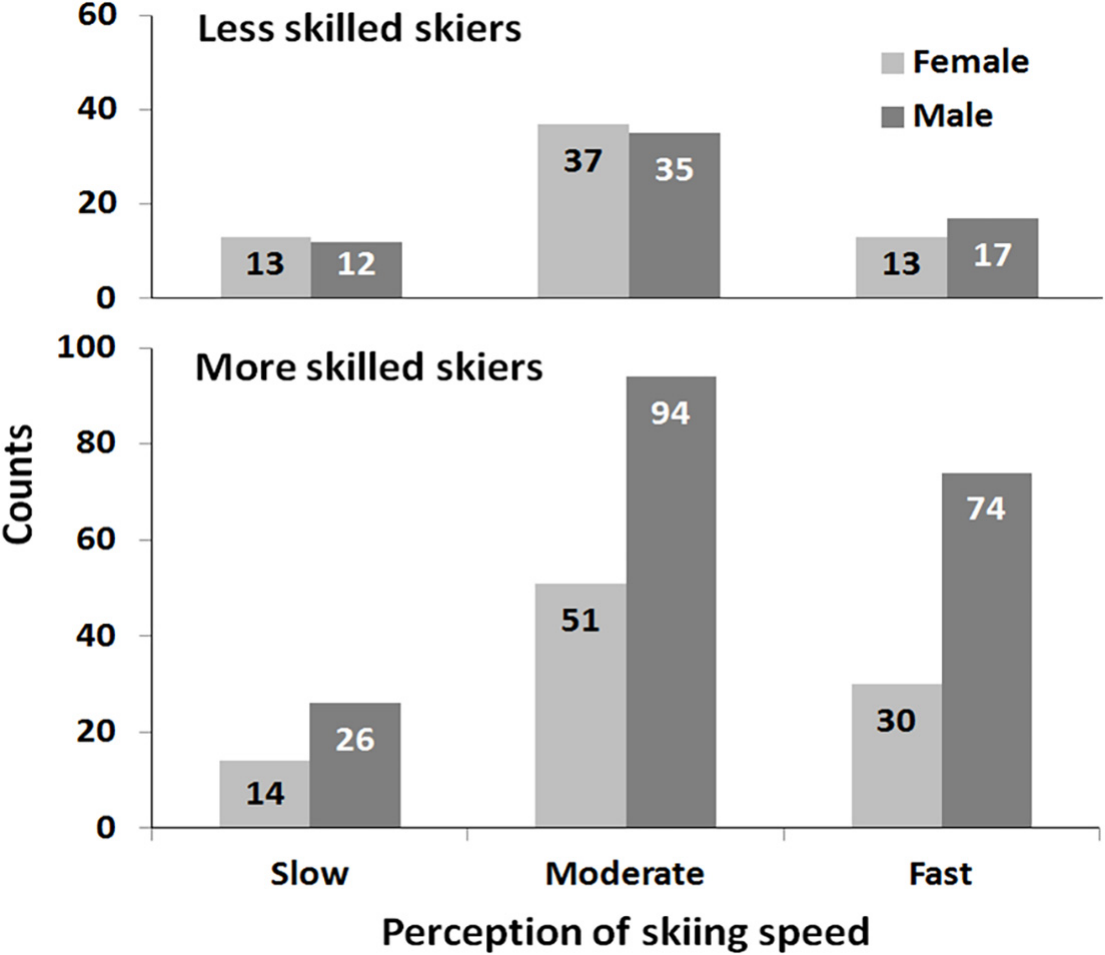
Numbers of male and female skiers according to skill level and perception of skiing speed.

Fig 2 shows the numbers of males and females and more skilled and less skilled skiers within the 3 speed perception groups. Resulting odds of males/females and odds ratios of more skilled/less skilled are shown in Table 1. No differences between 'slow', 'moderate' and 'fast' skiing speed perception groups were found regarding the ratio males to females neither in the less skilled (odds = 0.92 to 1.31) nor the more skilled skiers (odds = 1.86 to 2.47). The proportion of males was significantly larger in more skilled skiers (odds = 2.04) than in less skilled (odds = 1.02; OR = 2.01; p = .001). Within the sub-groups of perceived skiing speed the proportion of males was larger in the ‘moderate’ speed perceiving group (OR = 1.95, p = .022).

**Table 1:** Proportions male/female (Odds) and Odds ratios (OR) of more skilled/less skilled skiers with respect to perception of skiing speed.

	Total	Slow	Mod	Fast
All	1.63	1.41	1.47	2.12
Less skilled	1.02	0.92	0.95	1.31
More skilled	2.04	1.86	1.84	2.47
OR_more/less_	2.01	2.01	1.95	1.89
χ^2^ (fg = 1)	10.49	1.831	5.248	2.242
p	.001	.176	.022	.134

OR > 1 indicates a higher proportion of males in the more skilled group

### Measured skiing speed and speed perception

**Fig 3:**
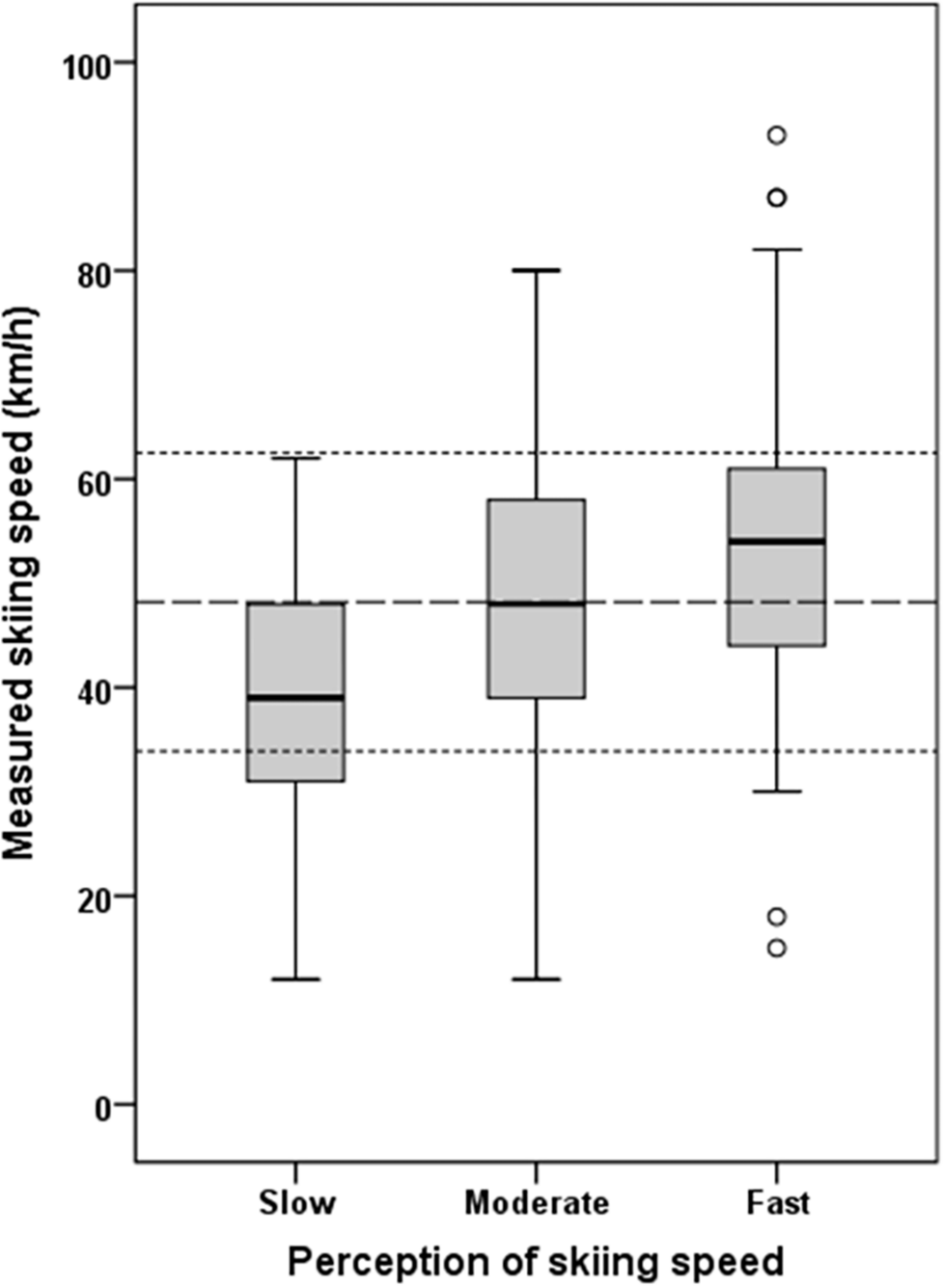
Measured skiing speeds according to their perception as ‘slow‘, ‘moderate‘ or ‘fast‘. Each Boxplot shows the values of Minimum, First quartile (Q_*1*_), Median, Third quartile (Q_*3*_) and Maximum; dashed lines show the Mean and the Standard deviation (SD).

The mean measured skiing speed of the cohort was 48.2±14.3 km/h (30.0±8.9 mph). In total, 15.6% of all skiers perceived their skiing speed as 'slow' with a mean speed of 39.4±12.2 km/h (24.5±7.6 mph), 52.2% as 'moderate' with a mean speed of 47.6±14.0 km/h (29.6±8.7 mph), and 32.2% as 'fast' with a mean speed of 53.5±13.7 km/h (33.2±8.5 mph) (Table 2; Fig 3), respectively.

**Table 2:** Measured skiing speed (km/h) according to speed perception and sex.

		Perception of skiing speed as		
Statistics (km/h)	Total (N = 416)	Slow (65)	Moderate (217)	Fast (134)
Minimum	12	12	12	15
Q_1_	39	31	39	44
Median	48	39	48	54
Q_3_	58	48	58	61
Maximum	93	62	80	93
Mean ± SD	48.2 ± 14.3	39.4 ± 12.2	47.6 ± 14.0	53.5 ± 13.7
Male	52.0 ± 14.1	42.0 ± 11.9	51.9 ± 13.4	56.2 ± 13.9
Female	42.1 ± 12.5	36.0 ± 11.7	41.1 ± 12.2	47.9 ± 11.3
2-way ANOVA:	Sex	F(1; 410) = 32.9	p < .001	η^2^ = .074
	Perc**e**ption	F(2; 410) = 21.4	p < .001	η^2^ = .094
	Interaction	F(2; 410) = 0.967	p < .385	η^2^ = .005

The differences of mean speeds between the three speed perception groups were significant (p < .001). However, the effect size was only medium due to the high variability of measured skiing speeds within the speed perception groups.

### The effect of sex, age, skiing skill level, risk taking behavior and helmet use on skiing speed

Average skiing speeds depending on the factors sex, age, skiing skill level, risk taking behavior and helmet use are shown in the left part of Fig 1 and in Table 3.

Results of factorial analyses considering these factors as main effects are shown in Table 4. Effect size on skiing speed was highest for sex (η^2^ = .071; p < .001, Fig 1) and resulted in a 10 km/h (6 mph) higher speed of males compared to females. Also, risk taking behavior and skill level had a small but significant effect on skiing speed (p = .003 and p = .05). More skilled and risk taking skiers skied on average 8 km/h (5 mph) faster, while ski helmet use and age had no effect (p = .12 and p = .55).

**Table 3:** Mean and SD of measured skiing speeds (km/h) of the 3 speed perception categories according to sex, age, skill level, risk taking behavior and helmet use.

		Perception of skiing Speed		
	Total	Slow	Moderate	Fast
Male	52.0 ± 14.1	42.0 ± 11.9	51.9 ± 13.4	56.2 ± 13.9
Female	42.1 ± 12.5	36.0 ± 11.7	41.1 ± 12.2	47.9 ± 11.3
≤ 30 yrs	49.7 ± 14.6	38.8 ± 10.5	49.2 ± 13.4	53.3 ± 15.1
31–40 yrs	48.9 ± 14.0	36.6 ± 14.4	48.1 ± 11.8	54.5 ± 13.9
41–50 yrs	49.2 ± 14.3	40.6 ± 11.1	49.2 ± 15.0	53.6 ± 12.7
> 50 yrs	44.9 ± 14.0	40.7 ± 13.1	44.1 ± 14.2	52.1 ± 11.8
More skilled	50.6 ± 14.4	40.1 ± 13.2	50.0 ± 14.2	55.6 ± 12.7
Less skilled	42.7 ± 12.6	38.6 ± 10.3	42.6 ± 12.0	46.4 ± 14.6
Risk taking	54.0 ± 13.1	47.0 ± 12.5	53.3 ± 11.8	56.7 ± 14.2
Cautious	46.3 ± 14.2	38.1 ± 11.7	45.7 ± 14.1	52.1 ± 13.3
Helmet used	49.3 ± 13.3	40.9 ± 10.9	48.4 ± 13.5	54.4 ± 11.8
Helmet not used	46.8 ± 15.5	37.4 ± 13.7	46.5 ± 14.5	52.2 ± 16.3

**Fig 4:**
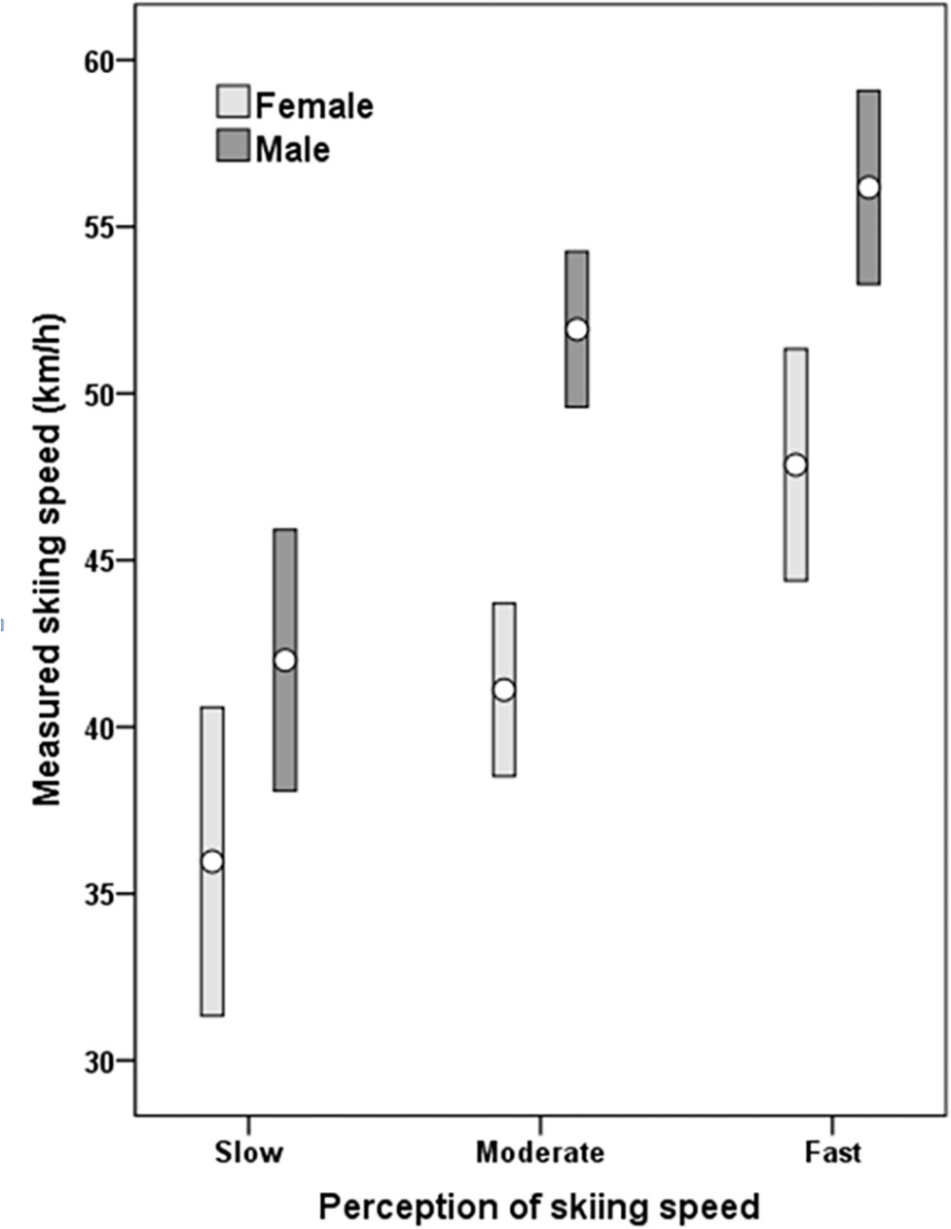
Mean and 95% Confidence interval (CI) of measured skiing speed of female and male skiers according to their perception of skiing speed as ‘slow‘,‘moderate‘ or ‘fast‘.

In Table 4 also the interaction effects of skiing speed perception on the one hand and sex, age, skill level, risk taking behavior and helmet use on skiing speed on the other hand are shown. For illustration of interaction, the variation of the differences between speed of males and females in the three perception groups is shown in Fig 4 (differences from 6 km/h to 10 km/h or 4 to 6 mph). The factor with most variation was skill level (differences between more and less skilled skiers from 1.5 km/h (0.9 mph) in the ‘slow’ group to 9.2 km/h (5.7 mph) in the ‘fast’ group; Table 3), but no factor interacted significantly with perception of skiing speed.

**Table 4:** Results of factorial ANOVA. The multifactorial model analyses the factors perception of skiing speed, sex, age, skiing skill level, risk taking behavior and helmet use as main effects and the 5 interaction effects between perception of skiing speed on the one hand and sex, age, skill level, risk taking behavior and helmet use on the other hand.

Main effects	df	F	p	η^2^
Perception of skiing speed	2	5.01	.007	.025
Sex	1	29.89	.000	.071
Age	3	0.71	.547	.005
Skill level	1	3.87	.050	.010
Risk taking behavior	1	8.85	.003	.022
Helmet use	1	2.40	.122	.006
Interaction effects of perception with				
Sex	2	0.92	.401	.005
Age	6	1.45	.196	.022
Skill level	2	1.96	.143	.010
Risk taking behavior	2	0.56	.574	.003
Helmet use	2	0.07	.932	.000
Error	389			
Total n = 413				
R^2^ = .295				

Notes: df Degrees of freedom

F F-statistic, ratio of the model variance to its error.

p Significance of difference between subgroups of a factor

η^2^ Effect size (partial η^2^). Amount of variation that is accounted by the factor.

R^2^ Amount of variation that is accounted by the model

## Discussion

The main goal of this study was to evaluate the actual skiing speed when skiers perceive their speed as slow, moderate or fast and how this depends on sex, age, and various other factors potentially influencing skiing speed of recreational skiers.

The main findings are that the perception of the individual skiing speed as slow, moderate or fast significantly depends on sex, skill level, and risk taking behavior. Males, more skilled skiers and risky skiers perceived their actual speed as fast when skiing significantly faster compared to females, less skilled and cautious skiers, respectively. Not surprisingly, mean measured speed differed significantly between the 3 speed perception groups with about 39 km/h (24 mph) for 'slow', 48 km/h (30 mph) for 'moderate', and 54 km/h (34 mph) for 'fast' skiing, respectively. Also, the measured maximum speed with 62 km/h (39 mph) for 'slow' skiing, 80 km/h (50 mph) for 'moderate', and 93 km/h (58 mph) for 'fast' skiing seems to clearly differentiate the three speed categories (Table 2). However, minimum speed for 'slow' and 'moderate' skiers was 12 km/h (8 mph) each, and for 'fast' skiers 15 km/h (9 mph) resulting in ranges of 50 km/h (31 mph) for ‘slow’ skiers, 68 km/h (42 mph) for ‘moderate’ and 78 km/h (49 mph) for ‘fast’ skiers, indicating that the perception of skiing speeds shows a huge variability. Also, due to the high variability of measured skiing speed within speed perception categories the effect size of speed perception was only medium, e.g. one quarter of 'slow' skiers skied faster than 48 km/h (30 mph) (which indicates the mean speed of the moderate group) while one quarter of 'fast' skiers skied slower than 44 km/h (27 mph) (Table 2), indicating that an individual perception of skiing speed is a less reliable method.

Sex was found to have a small but significant effect on speed (η^2^ = .071) and the mean speed of the three speed categories differed by about 6–10 km/h (4–6 mph) between sexes. In other words, 'slow' or 'moderate' or 'fast' skiing does not mean the same among male and female skiers. According to the ISO 11088 standard for binding release values [9], skiers have to classify themselves into one out of three skiing types by differentiating between skiing speed ('slow to moderate' vs. 'fast'), terrain (‘gentle to moderate’ vs. ‘steep’) and skiing style (‘cautious’ vs. ‘aggressive’). Let’s assume a male and a female of equal age, height, and weight and of equal ski shoe sole length also both classified themselves as type-3 skier (fast speed, steep terrain, aggressive style). Both of them would get the same binding setting values without considering any sex factor. However, according to the results of this study, female skiers may perceive their actual speed faster than male skiers which would result in a higher binding setting value of the female skier requiring a higher torque to release the binding during a fall. Studies on knee injured skiers reported a binding non-release in 74–88% of female skiers compared to 55–67% of male skiers [10, 14, 15]. There seems evidence that female skiers have a higher amount of falls with binding non-release although sexes seem not to differ regarding the date of the last binding adjustment [10,16], nor regarding deviations from recommended z-values according the ISO 11088 standard [16], nor with regard to the type of fall leading to an ACL injury [10]. In addition, in a recent study injured females reported a significantly higher amount of binding non-releases compared to injured males (51 vs. 32%), irrespective of injured body part [17]. One might suspect that compared to a ‘slow to moderate’ or ‘fast’ skiing male the binding setting for a ‘slow to moderate’ or ‘fast’ skiing female is too high resulting in a higher amount of binding non-releases. Thus, speed perception should also be considered as a potential cause of injury.

Therefore, based on our results, we strongly would recommend considering a sex factor within the ISO 11088 standard for binding values. Despite sex, also skill level (η^2^ = .010) and risk taking behaviour (η^2^ = .022) are small but significant factors associated with the perception of skiing speed. More skilled and risk taking skiers showed about 8 km/h higher mean speeds compared to less skilled and cautious skiers (Table 3). Whereas skiing skills and risk taking behaviour are already considered in the ISO 11088 standard sex is not. However, the actual speed differs significantly between male and female skiers perceiving the same skiing speed.

It seems notable that like prior studies [11, 18], helmet use was not associated with speed or speed perception casting doubt on the risk compensation hypothesis which means that ski helmet use has lowered the individual risk level and skiers using a helmet are trying to change their behaviour to bring them back to their target risk level by skiing faster or more aggressively or on more difficult runs [19].

A few limitations have to be considered when interpreting our results. Firstly, answering questions about oneself might lead to under reporting or over reporting of health-risk behaviors affected by cognitive and situational factors [12, 20]. Secondly, speed measurements on ski slopes with a radar speed might be less accurately compared to speed measurements with GPS [21] although Shealy et al. [6] verified radar speed gun measurements by comparison with a GPS device.

In conclusion, the perception of skiing speed as fast, moderate or slow depends on sex, skill level, and risk taking behaviour. Males, more skilled skiers and risky skiers perceived their actual speed as fast, moderate and slow, when skiing significantly faster compared to females, less skilled and cautious skiers, respectively. These findings should be considered when discussing the introduction of a sex factor into the ISO 11088 standard for setting binding release values.
