# Correction: Factors Associated with the Perception of Speed among Recreational Skiers

**DOI:** 10.1371/journal.pone.0135763

**Published:** 2015-08-10

**Authors:** The PLOS ONE Staff

## Notice of Republication

This article was republished on July 29, 2015, to correct errors in equations that were introduced during the typesetting process. Please download this article again to view the correct version. The originally published, uncorrected article and the republished, corrected article are provided here for reference. The publisher apologizes for the error.

## Supporting Information

S1 File Originally published, uncorrected article. (PDF)

S2 File Republished corrected article. (PDF)
